# Quantitative promoter methylation differentiates carcinoma ex pleomorphic adenoma from pleomorphic salivary adenoma

**DOI:** 10.1038/sj.bjc.6605953

**Published:** 2010-11-09

**Authors:** A G Schache, G Hall, J A Woolgar, G Nikolaidis, A Triantafyllou, D Lowe, J M Risk, R J Shaw, T Liloglou

**Affiliations:** 1 Department of Molecular & Clinical Cancer Medicine, Institute of Translational Medicine, University of Liverpool, Daulby Street, Liverpool L69 3GN, UK; 2 Regional Maxillofacial Unit, University Hospital Aintree, Longmoor Lane, Liverpool L9 7AL, UK; 3 Department of Histopathology, University of Manchester, Oxford Road, Manchester M13 9WL, UK; 4 Department of Oral Pathology, University of Liverpool, Pembroke Place, Liverpool L3 5PS, UK; 5 Evidence-based Practice Research Centre, Edge Hill University, St Helens Road, Ormskirk, Lancashire L39 4QP, UK

**Keywords:** carcinoma ex pleomorphic adenoma, methylation, salivary gland tumour

## Abstract

**Background:**

Potential epigenetic biomarkers for malignant transformation to carcinoma ex pleomorphic adenoma (Ca ex PSA) have been sought previously with and without specific comparison with the benign variant, pleomorphic salivary adenoma (PSA). Previous analysis has been limited by a non-quantitative approach. We sought to demonstrate quantitative promoter methylation across a panel of tumour suppressor genes (TSGs) in both Ca ex PSA and PSA.

**Methods:**

Quantitative methylation-specific real-time polymerase chain reaction (qMSP) analysis of p16^INK4A^, CYGB, RASSF1, RAR*β*, human telomerase reverse transcriptase (hTERT), Wilms’ tumour 1 (WT1) and TMEFF2 gene promoters was undertaken on bisulphite-converted DNA, previously extracted from archival fixed tissue specimens of 31 Ca ex PSA and an unrelated cohort of 28 PSA. All target regions examined had formerly been shown to be hypermethylated in salivary and/or mucosal head and neck malignancies.

**Results:**

The qMSP demonstrated abnormal methylation of at least one target in 20 out of 31 (64.5%) Ca ex PSA and 2 out of 28 (7.1%) PSA samples (*P*<0.001). RASSF1 was the single gene promoter for which methylation is shown to be a statistically significant predictor of malignant disease (*P*<0.001) with a sensitivity of 51.6% and a specificity of 92.9%. RAR*β*, TMEFF2 and CYGB displayed no apparent methylation, while a combinatory epigenotype based on p16, hTERT, RASSF1 and WT1 was associated with a significantly higher chance of detecting malignancy in any positive sample (odds ratio: 24, 95% CI: 4.7–125, *P*<0.001).

**Conclusions:**

We demonstrate the successful application of qMSP to a large series of historical Ca ex PSA samples and report on a panel of TSGs with significant differences in their methylation profiles between benign and malignant variants of pleomorphic salivary adenoma. qMSP analysis could be developed as a useful clinical tool to differentiate between Ca ex PSA and its benign precursor.

Carcinoma ex pleomorphic adenoma (Ca ex PSA) is a rare and poorly understood malignancy of salivary glands. It accounts for 3–4% of salivary neoplasms and approximately 12% of all salivary malignancies (Gnepp, 1991). It has been suggested that Ca ex PSA originates as a result of malignant transformation of ductal epithelial and/or abluminal myoepithelial cells within pre-existing pleomorphic salivary adenomas (PSA), the majority of which are untreated or recurrent in nature (Eneroth and Zetterberg, 1974). These cancers are typically high grade in nature, with frequent metastasis, and have a poor prognosis (Lewis *et al*, 2001; Olsen and Lewis, 2001).

Quantifying the risk of malignant transformation in PSA has not been possible; however, there appears to be a temporal relationship such that long-standing tumours have higher risk for malignant transformation. This risk increases from 1.6% for tumours present <5 years to 9.6% for PSA present in >15 years (Ellis, 1996).

Understanding of the mechanisms underlying malignant transformation from PSA to Ca ex PSA has been restricted by a paucity of available tissue. A variety of molecular mechanisms and potential biomarkers have been suggested in the context of various salivary malignancies, with studies exploring differential expression of cyclin D1, p16, p53, EGFR, TGF*α*, Ki-67 and Mcm-2 by immunohistochemistry (Lewis *et al*, 2001; Augello *et al*, 2006; Katori *et al*, 2007; Patel *et al*, 2007; Vargas *et al*, 2008). The role of DNA-based biomarkers has not been extensively explored in this setting. Individual mutations are generally uncommon; however, the encouraging specificity and sensitivity of epigenetic biomarkers has encouraged some studies comparing series of benign and malignant salivary tumours (Maruya *et al*, 2004; Williams *et al*, 2006; Lee *et al*, 2008). Earlier work was limited by the non-quantitative methylation-specific real-time polymerase chain reaction (MSP) techniques available (Maruya *et al*, 2003). The merit of quantitative assays for promoter methylation status lies in their ability to establish thresholds in the assessment of methylation/non-methylation and potentially the classification of benign and malignant counterparts. The case for pyrosequencing methylation analysis (PMA) in salivary (Lee *et al*, 2008) and squamous mucosal malignancy of the head and neck (Shaw *et al*, 2006) has been made when fresh frozen tissue is available. In the case of archival samples in less common tumours, it is more likely that fixed formalin paraffin-embedded (FFPE) tissue will be available. In such a situation the reduced integrity of DNA available may restrict the potential advantages of PMA, while alternative quantitative techniques such as real-time MSP (RT–MSP) (Eads) may have a role (Lee *et al*, 2008).

An evaluation of tumour suppressor gene (TSG) promoter hypermethylation limited to Ca ex PSA and its benign precursor has not previously been performed. Such analysis may offer a potential biomarker for progression, the basis for a diagnostic tool, or further insight into the molecular mechanisms responsible. We evaluate a large archival series of Ca ex PSA cases alongside unrelated PSA using RT–polymerase chain reaction (PCR) to quantify promoter methylation in seven TSGs previously shown to be methylated in salivary and in epithelial malignancies, including those of salivary origin.

## Materials and Methods

### Case selection

The archives of the oral pathology laboratories of the Liverpool and Manchester University Hospital Dental Schools were searched using SNOMED code (M89413). Electronic databases were searched from 1993 to 2007. Diagnosis was confirmed through review of all potential H&E stained slides and cases with a demonstrable pre-existing PSA or its ‘ghost’ were included in the study (*n*=24). Additional cases (*n*=7) were also included in which the clinical presentation indicated Ca ex PSA: history of a long-standing stable swelling with recent rapid alteration in size and with histological confirmation of a malignant tumour, usually high grade and often showing multiple patterns of differentiation. Presence of multiple patterns of malignant salivary gland type tumours or diverse differentiation alone was not accepted as definite evidence of Ca ex PSA. All cases were reviewed independently by two pathologists (GH, JAW). Cases of PSA were identified by database searching (SNOMED code M89400) (*n*=28).

Available FFPE blocks from the selected cases were retrieved. The human tissue that formed the basis for this research was utilised under previously granted ethical approval (Central Liverpool) LREC 06/Q1505/71.

### DNA extraction and bisulphite treatment

DNA extraction was undertaken using a modification of the method reported by Banerjee *et al* (1995) for reasons of anticipated reduced yield (complete details are available on request). The EZ DNA Methylation kit (Zymo Research Corporation, Orange, CA, USA) was used to bisulphite treat 2 *μ*g of previously prepared DNA. The treated DNA was eluted in 50 *μ*l of 0.1 × TE buffer.

### qMSP analysis

Quantitative methylation-specific real-time polymerase chain reaction was used to determine TSG methylation in each of the samples. The gene promoters for p16^INK4A^, CYGB, RASSF1, RAR*β*, human telomerase reverse transcriptase (hTERT), Wilms’ tumour 1 (WT1) and TMEFF2 were included in the panel for methylation detection. The qMSP assays were designed using Primer Express 3.0 software (Applied Biosystems, Foster City, CA, USA). The primer and probe sequences and PCR conditions used for these real-time assays are given in Tables 1 and 2. A total reaction volume of 25 *μ*l in each reaction contained Taqman Universal Master Mix II (Applied Biosystems), 500 nM of each primer, 250 nM of probe and 100 ng of bisulphite-treated DNA. A separate assay utilising a methylation-independent primer/probe set on the *β*-actin gene (ACTB) was used to normalise for the DNA input in each sample. Real-time PCR reactions were performed on an Applied Biosystems 7500 FAST system. Serial dilutions (80–5%) of *in vitro* methylated (SssI) human lymphocyte DNA made in untreated lymphocyte DNA were used as a reference. ΔΔCT values were generated for each target after normalisation by ACTB values. The RQ values were subsequently calculated (2^−ΔΔCT^) and referenced to the artificially methylated samples for statistical analysis. The associations between sample methylation and tumour type (Ca ex PSA *vs* PSA) were tabulated, and Fisher's exact test was used to measure their significance. Duplicate reactions were carried out for p16^INK4A^, hTERT, WT1 and RASSF1. Determination of positive results following duplicate reactions for individual samples is detailed below.

## Results

A threshold of 5% methylation was used to define positive methylation above which a sample was deemed to be methylated at that particular gene promoter. This was based on our previous work for the minimum levels that are both biologically meaningful and functionally relevant in non-microdissected tissues (Shaw *et al*, 2006).

Duplicate experiments were undertaken for those gene promoters demonstrating methylation in this sample set and each sample was only deemed to be positive if methylation levels were above 5%. Where conflict existed between runs, the average methylation level was utilised as a final arbiter (Table 3A and B).

**Figure 1:**
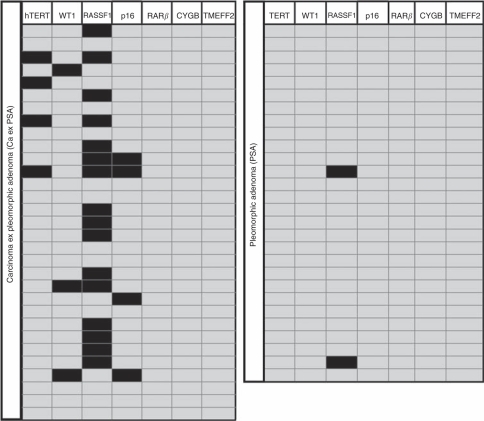
qMSP results. Representation of qMSP data for Ca ex PSA (left) and PSA (right) samples. ▪, Promoter methylation positive; 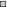 Promoter methylation negative (at 5% methylation threshold). Data from hTERT, WT1, RASSF1 and p16 data is from duplicate experiments.

Promoter methylation was observed in four of the seven gene promoters included on the methylation panel (Figure 1). Methylation was demonstrated in a total of 20 out of 31 Ca ex PSA samples (64.5%) and 2 out of 28 (7.1%) PSA samples (*P*<0.001). The hTERT, WT1 and p16 ^INK4A^ methylation showed 12.9%, 9.7% and 12.9% sensitivity, respectively, and 100% specificity (Table 4). RAR*β*, CYGB and TMEFF2 methylation was not apparent in any sample (Figure 1). RASSF1 was the single gene promoter for which methylation is shown to be a statistically significant predictor of malignant disease (*P*<0.001) with a sensitivity of 51.6% and a specificity of 92.9% (Table 4).

When aggregation of the results is undertaken, the presence of methylation above the 5% threshold in hTERT, WT1, RASSF1 or p16 ^INK4A^ was associated with a significantly higher chance of an individual sample having malignant pathology (odds ratio: 24, 95% CI: 4.7–125, *P*>0.001). As a panel, the sensitivity for detecting malignancy for any positive assay was 64.5% (95% CI: 45–81%) and specificity for excluding benign disease was 92.9% (95% CI: 77–99%).

To determine whether positive methylation was occurring in an interrelated manner, a goodness of fit calculation of observed *vs* expected methylation was undertaken. No significant concordance was apparent (*χ*^2^=0.16, 2df; *P*=0.92).

## Discussion

This study provides for the first time a comparative DNA methylation profiling between Ca ex PSA and PSA. Owing to the rarity of Ca ex PSA, the investigation was based on only 31 cases of Ca ex PSA, even though the combined cases of two large British cities constituting a population of around 7 million over 14 years were used. Seven cases were included based on particular clinical history and histopathological evidence of malignancy rather than the histologically demonstrable presence of pre-existing/ghost of PSA. It is noted that these cases gave comparable results with those that did present with demonstrable PSA, which supports their inclusion in the material.

Significant tumour-specific promoter methylation (for Ca ex PSA) is apparent at RASSF1 by comparison with PSA. The remaining promoters examined here, did not display, on a single marker basis, statistically significant variation between tumour types. However, a combinatory epigenotype consisting of hTERT, WT1, RASSF1 and p16^INK4A^ (at least one positive) demonstrated a positive predictive value for malignancy.

The concept of a CpG island methylator phenotype (CIMP) as described in oral squamous mucosal cancer (Shaw *et al*, 2007) was not apparent within this study as hypermethylation of individual promoters occurs in a non-interrelated or independent manner. It will though require a larger study incorporating a larger number of promoters to substantiate the real frequency of CIMP in this type of malignancy.

The high sensitivity (51.6%) of RASSF1 to detect malignant disease is combined with an imperfect, albeit still high specificity (92.9%). At this point, it is not clear whether this 7.1% of RASSF1 methylation-positive PSA cases represent false positives or benign lesions with an elevated potential towards malignant transition to Ca ex PSA. As none of these benign lesions were managed with observation only, their malignant potential cannot be evaluated. This possibility was alluded to by Williams *et al* (2006) in their description of the role of TSG hypermethylation in salivary gland tumourigenesis. This series reported 8.7% cases with methylation in benign tumours compared with 27% of malignant cases, although no data on Ca ex PSA was available. Lee *et al* (2008), in their comparative analysis of methylation techniques, found RASSF1 to be methylated in 35% of 69 malignant salivary tumours and 50% of Ca ex PSA, although only six cases were available. RASSF1 (Ras association domain-containing protein 1) gene encodes a protein similar to the RAS effector proteins. The encoded protein interacts with DNA repair protein XPA and inhibits the accumulation of cyclin D1, thus inducing cell cycle arrest (Shivakumar *et al*, 2002). Loss or altered expression of this gene has been associated with the pathogenesis and progression of a variety of cancers (Burbee *et al*, 2001; Donninger *et al*, 2007; Buckingham *et al*, 2010) including oral SCC (Ghosh *et al*, 2008; Huang *et al*, 2009).

There are no reported studies exploring quantitative methylation of p16^INK4A^ in salivary malignancy, which is surprising as it appears to be implicated in a wide variety of cancer types, often offering a high degree of specificity. We have previously shown that p16^INK4A^ is hypermethylated in 28% of oral squamous cell carcinomas (OSCCs) compared with 4% of surrounding unaffected margins (Shaw *et al*, 2006). In addition, p16^INK4A^ promoter methylation was detected in 57% of oral epithelial dysplasias undergoing malignancy in comparison with 8% of those which did not transform (Hall *et al*, 2008). In this study, p16^INK4A^ offered 100% specificity and 12.9% sensitivity in determining Ca ex PSA from PSA. A previous study reported p16^INK4A^ hypermethylation in 14% of a series of 28 PSA and 100% in five salivary carcinomas, one of which was Ca ex PSA (Augello *et al*, 2006). This study used a non-quantitative MSP approach, most probably reflecting the higher rate of positives. The need for quantitative assays in the determination of clinically meaningful threshold values is widely accepted to date.

Human telomerase reverse transcriptase is a component of the telomerase ribonucleoprotein complex and is tightly regulated, such that it is not normally detectable in the majority of somatic cells. As almost all human cancer cells express telomerase while most normal cells do not, the hTERT promoter has been characterised as a molecular switch for the selective expression of target genes in tumour cells (Takakura *et al*, 1999). Within our cohort of salivary tumours, hTERT promoter methylation was apparent in only 12.9% of Ca ex PSA and absent in PSA. Previous reference to hTERT activity in head and neck cancer has been with respect to OSCC and related mucosal dysplasia. In particular, using RT–PCR expression analysis, Pannone *et al* (2007) found 66% of OSCC tumours to be overexpressing hTERT by comparison with normal tissues. The hTERT promoter hypermethylation has been implicated in cervical cancer progression while not correlating with down-regulation hTERT expression (Widschwendter *et al*, 2004; Iliopoulos *et al*, 2009; Kumari *et al*, 2009). It has been postulated that methyl-CpG-binding domain proteins aberrations may be responsible for this paradox (Chatagnon *et al*, 2009).

Wilms’ tumour 1 gene encodes for a protein essential for normal urogenital development, but it has been shown to be overexpressed in several epithelial tumours and tumour cell lines including head and neck squamous cell carcinoma (Oji *et al*, 1999, 2003). Abnormal expression of WT1 was shown in 7% of benign and 31% of malignant salivary tumours (Nagel *et al*, 2003), none of which were Ca ex PSA, while expression was not detected in normal adjacent salivary tissue. Conversely, WT1 gene hypermethylation has been observed in cervical and colorectal cancer and has been shown to correlate with reduced WT1 expression in ovarian clear cell carcinoma (Kaneuchi *et al*, 2005; Lai *et al*, 2008). Furthermore, our preliminary methylation array analysis on oral SCC has identified WT1 among potential prognostic biomarkers (unpublished data). Thus, the control of the WT1 gene may differ in cancers from different sites. In the present investigation, analysis of WT1 promoter methylation failed to demonstrate methylation in any PSA samples and <10% of Ca ex PSA samples were methylated at this promoter. The utility of this biomarker in salivary cancer is thus limited to its use in a panel.

Our investigation further demonstrates the ability to gain quantitative methylation data from archival samples. Despite the acknowledged limitations in DNA quality from archival material, qMSP successfully provided reliable and reproducible results across several assays.

Most individuals presenting with PSA are advised to undergo resection of the tumour to exclude the relative risk of malignant transformation. A marker for malignant progression to Ca ex PSA from PSA would be of significant benefit to these individuals as any risk would, therefore, become quantifiable.

At present, fine needle aspirate cytology (FNAC) is a major component in the diagnosis of salivary tumours, particularly those arising in the parotid gland. Klijanienko *et al* (1999) explored the diagnostic pitfalls and considerations of this technique in Ca ex PSA and found that although it was accurate in diagnosis of histologically high-grade Ca ex PSA, sensitivity and specificity fell in lower-grade Ca ex PSA. To avoid these diagnostic difficulties, it is possible to envisage a panel of genes applied as a useful adjunct to such FNAC samples or equally to core biopsies, which have been impossible to diagnose or grade on histological grounds alone. The results of this study highlight significant differences in methylation between the benign and malignant tumour type and a broader array of validated methylation biomarkers might constitute a valuable diagnostic tool.

With less constrained resources, in particular original sample DNA, innovative techniques such as methylation microarray technology could be used to highlight further targets worthy of investigation and validation as part of a more extensive panel of TSGs. It is, however, unlikely that a comprehensive and specific methylation panel could ever be economically validated for such a rare tumour site such as Ca ex PSA; however, research in more common tumour sites may add significantly to this process. As an example, the impressive sensitivity and specificity of GSTP1 methylation in prostate adenocarcinoma (Jeronimo *et al*, 2001) has led to a variety of studies in its clinical use.

In common with other clinical sites, the management of benign salivary neoplasms includes complex surgery in an attempt to avoid the unpredictable risks of future malignant transformation. It is hoped that the accumulation and validation of data such as is presented here might allow a more conservative approach in the future. Such validation of a methylation biomarker panel may, therefore, have diagnostic, prognostic and therapeutic importance in the management of both the benign and malignant variants of this salivary tumour.

## Figures and Tables

**Table 1:** qMSP primer and probe sequences

**Target**	**F sequence**	**R sequence**	**Probe sequence**
TMEFF2	GGAGAGTTAAGGCGTTTCGTAGTTC	CCGACTACCTCTTCCCACGTAA	CGAACGAACTAAAAAC
hTERT	TTGGGAGTTCGGTTTGGTTTC	CACCCTAAAAACGCGAACGA	AGCGTAGTTGTTTCGG
WT1	GAGGAGTTAGGAGGTTCGGTC	CACCCCAACTACGAAAACG	AGTTCGGTTAGGTAGC
RAR*β*	GAGGATTGGGATGTCGAGAAC	CTTACAAAAAACCTTCCGAATACG	AGCGATTCGAGTAGGGT
RASSF1	TATTTTCGCGTGGTGTTTTGC	CCCTTCCTTCCCTCCTTCG	TCGTTGTGGTCGTTCG
CYGB	GTGTAATTTCGTCGTGGTTTGC	CCGACAAAATAAAAACTACGCG	TGGGCGGGCGGTAG
P16^INK4A^	GGAGGGGGTTTTTTCGTTAGTATC	CTACCTACTCTCCCCCTCTCCG	AACGCACGCGATCC
Actin *β*	GGGTGGTGATGGAGGAGGTT	TAACCACCACCCAACACACAAT	TGGATTGTGAATTTGTGTTTG

Abbreviations: hTERT=human telomerase reverse transcriptase; qMSP=quantitative methylation-specific real-time polymerase chain reaction; WT1=Wilms’ tumour 1.

Details of forward (F), reverse (R) and probe sequences for each of the seven tumour suppressor genes analysed and for actin *β*.

**Table 2:** qMSP PCR conditions

**Target**	**Melting**	**Annealing**	**Extension**
TMEFF2	95°C, 15 s	58°C, 20 s	60°C, 40 s
hTERT	95°C, 15 s	65°C, 5 s	62°C, 45 s
WT1	95°C, 15 s	62°C, 60 s	
RAR*β*	95°C, 30 s	65°C, 5 s	62°C, 45 s
RASSF1	95°C, 15 s	60°C, 60 s	
CYGB	95°C, 15 s	64°C, 5 s	61°C, 45 s
P16^INK4A^	95°C, 15 s	60°C, 60 s	
Actin *β*	95°C, 15 s	58°C, 20 s	60°C, 40 s

Abbreviations: hTERT=human telomerase reverse transcriptase; PCR=polymerase chain reaction; qMSP=quantitative methylation-specific real-time polymerase chain reaction; WT1=Wilms’ tumour 1.

Details of PCR conditions for each of the seven tumour suppressor genes analysed and for actin *β*.

**Table 3:** Average ΔΔCT and RQ for each (a) Ca ex PSA sample and (b) PSA sample

	**hTERT**		**WT1**		**RASSF1**		**p16**		**RAR*β***		**CYGB**		**TMEFF2**	
**Sample**	**ΔΔCT**	**RQ**	**ΔΔCT**	**RQ**	**ΔΔCT**	**RQ**	**ΔΔCT**	**RQ**	**ΔΔCT**	**RQ**	**ΔΔCT**	**RQ**	**ΔΔCT**	**RQ**
**5% Control**	**7.9**	**1.0**	**5.6**	**1.0**	**5.2**	**1.0**	**5.7**	**1.0**	**9.5**	**1.0**	**4.5**	**1.0**	**6.2**	**1.0**
(A) Ca ex PSA	0.0	0.0	0.0	0.0	1.4	14.2	0.0	0.0	0.0	0.0	0.0	0.0	0.0	0.0
	9.2	0.4	0.0	0.0	0.0	0.0	0.0	0.0	0.0	0.0	0.0	0.0	0.0	0.0
	6.9	2.0	0.0	0.0	1.1	16.7	0.0	0.0	0.0	0.0	0.0	0.0	0.0	0.0
	0.0	0.0	5.3	1.3	0.0	0.0	0.0	0.0	0.0	0.0	0.0	0.0	0.0	0.0
	3.7	18.2	0.0	0.0	0.0	0.0	0.0	0.0	0.0	0.0	0.0	0.0	0.0	0.0
	8.1	0.9	0.0	0.0	5.2	1.0	0.0	0.0	0.0	0.0	0.0	0.0	0.0	0.0
	10.5	0.2	0.0	0.0	8.6	0.1	0.0	0.0	0.0	0.0	0.0	0.0	0.0	0.0
	5.6	4.9	0.0	0.0	1.2	15.8	0.0	0.0	0.0	0.0	0.0	0.0	0.0	0.0
	0.0	0.0	0.0	0.0	0.0	0.0	0.0	0.0	0.0	0.0	0.0	0.0	0.0	0.0
	0.0	0.0	0.0	0.0	5.3	0.9	0.0	0.0	0.0	0.0	0.0	0.0	0.0	0.0
	10.5	0.2	0.0	0.0	2.9	4.9	4.2	2.9	0.0	0.0	0.0	0.0	0.0	0.0
	7.7	1.1	8.0	0.2	3.9	2.4	5.0	1.6	0.0	0.0	0.0	0.0	0.0	0.0
	9.4	0.4	8.7	0.1	3.1	4.3	0.0	0.0	0.0	0.0	0.0	0.0	0.0	0.0
	0.0	0.0	8.5	0.1	8.0	0.1	0.0	0.0	0.0	0.0	0.0	0.0	0.0	0.0
	9.6	0.3	10.0	0.0	2.9	4.9	0.0	0.0	0.0	0.0	0.0	0.0	0.0	0.0
	12.8	0.0	0.0	0.0	4.1	2.2	0.0	0.0	0.0	0.0	0.0	0.0	0.0	0.0
	10.5	0.2	0.0	0.0	2.1	8.8	0.0	0.0	0.0	0.0	0.0	0.0	0.0	0.0
	10.7	0.1	0.0	0.0	0.0	0.0	0.0	0.0	0.0	0.0	0.0	0.0	0.0	0.0
	9.7	0.3	0.0	0.0	0.0	0.0	0.0	0.0	0.0	0.0	0.0	0.0	0.0	0.0
	12.5	0.0	0.0	0.0	4.4	1.8	0.0	0.0	0.0	0.0	0.0	0.0	0.0	0.0
	0.0	0.0	4.3	2.5	4.9	1.2	0.0	0.0	0.0	0.0	0.0	0.0	0.0	0.0
	8.9	0.5	0.0	0.0	5.2	1.0	4.1	3.0	0.0	0.0	0.0	0.0	0.0	0.0
	8.5	0.6	7.3	0.3	0.0	0.0	0.0	0.0	0.0	0.0	0.0	0.0	0.0	0.0
	8.2	0.8	6.1	0.7	3.3	3.7	0.0	0.0	0.0	0.0	0.0	0.0	0.0	0.0
	0.0	0.0	6.5	0.5	4.3	1.9	0.0	0.0	0.0	0.0	0.0	0.0	0.0	0.0
	0.0	0.0	0.0	0.0	3.6	2.9	6.8	0.5	0.0	0.0	0.0	0.0	0.0	0.0
	13.8	0.0	10.0	0.0	5.0	1.1	0.0	0.0	0.0	0.0	0.0	0.0	0.0	0.0
	11.8	0.1	4.1	2.9	0.0	0.0	5.1	1.5	0.0	0.0	0.0	0.0	0.0	0.0
	0.0	0.0	0.0	0.0	0.0	0.0	0.0	0.0	0.0	0.0	0.0	0.0	0.0	0.0
	0.0	0.0	0.0	0.0	0.0	0.0	0.0	0.0	0.0	0.0	0.0	0.0	0.0	0.0
	12.9	0.0	0.0	0.0	4.4	1.7	0.0	0.0	0.0	0.0	0.0	0.0	0.0	0.0
														
(B) PSA	0.0	0.0	0.0	0.0	0.0	0.0	0.0	0.0	0.0	0.0	0.0	0.0	0.0	0.0
	15.5	0.0	0.0	0.0	0.0	0.0	0.0	0.0	0.0	0.0	0.0	0.0	0.0	0.0
	0.0	0.0	0.0	0.0	0.0	0.0	0.0	0.0	0.0	0.0	0.0	0.0	0.0	0.0
	0.0	0.0	0.0	0.0	12.5	0.0	0.0	0.0	0.0	0.0	0.0	0.0	0.0	0.0
	11.0	0.1	0.0	0.0	0.0	0.0	0.0	0.0	0.0	0.0	0.0	0.0	0.0	0.0
	9.9	0.2	0.0	0.0	12.2	0.0	0.0	0.0	0.0	0.0	0.0	0.0	0.0	0.0
	0.0	0.0	0.0	0.0	9.0	0.1	0.0	0.0	0.0	0.0	0.0	0.0	0.0	0.0
	10.3	0.2	0.0	0.0	12.0	0.0	0.0	0.0	0.0	0.0	0.0	0.0	0.0	0.0
	0.0	0.0	0.0	0.0	10.7	0.0	0.0	0.0	0.0	0.0	0.0	0.0	0.0	0.0
	0.0	0.0	0.0	0.0	0.0	0.0	0.0	0.0	0.0	0.0	0.0	0.0	0.0	0.0
	0.0	0.0	0.0	0.0	5.3	0.9	0.0	0.0	0.0	0.0	0.0	0.0	0.0	0.0
	0.0	0.0	0.0	0.0	5.1	1.1	0.0	0.0	0.0	0.0	0.0	0.0	0.0	0.0
	0.0	0.0	0.0	0.0	0.0	0.0	0.0	0.0	0.0	0.0	0.0	0.0	0.0	0.0
	0.0	0.0	0.0	0.0	0.0	0.0	0.0	0.0	0.0	0.0	0.0	0.0	0.0	0.0
	0.0	0.0	0.0	0.0	8.4	0.1	0.0	0.0	0.0	0.0	0.0	0.0	0.0	0.0
	14.1	0.0	0.0	0.0	7.3	0.2	0.0	0.0	0.0	0.0	0.0	0.0	0.0	0.0
	0.0	0.0	0.0	0.0	8.4	0.1	0.0	0.0	0.0	0.0	0.0	0.0	0.0	0.0
	0.0	0.0	0.0	0.0	11.9	0.0	0.0	0.0	0.0	0.0	0.0	0.0	0.0	0.0
	0.0	0.0	0.0	0.0	0.0	0.0	0.0	0.0	0.0	0.0	0.0	0.0	0.0	0.0
	0.0	0.0	0.0	0.0	0.0	0.0	0.0	0.0	0.0	0.0	0.0	0.0	0.0	0.0
	0.0	0.0	0.0	0.0	0.0	0.0	0.0	0.0	0.0	0.0	0.0	0.0	0.0	0.0
	0.0	0.0	0.0	0.0	5.6	0.8	0.0	0.0	0.0	0.0	0.0	0.0	0.0	0.0
	0.0	0.0	0.0	0.0	11.7	0.0	0.0	0.0	0.0	0.0	0.0	0.0	0.0	0.0
	12.3	0.0	0.0	0.0	0.0	0.0	0.0	0.0	0.0	0.0	0.0	0.0	0.0	0.0
	0.0	0.0	0.0	0.0	12.3	0.0	0.0	0.0	0.0	0.0	0.0	0.0	0.0	0.0
	0.0	0.0	0.0	0.0	0.0	0.0	0.0	0.0	0.0	0.0	0.0	0.0	0.0	0.0
	0.0	0.0	0.0	0.0	4.5	1.6	0.0	0.0	0.0	0.0	0.0	0.0	0.0	0.0
	16.8	0.0	8.1	0.2	6.1	0.5	0.0	0.0	0.0	0.0	0.0	0.0	0.0	0.0

Abbreviations: Ca ex PSA=carcinoma ex pleomorphic adenoma; hTERT=human telomerase reverse transcriptase; PSA=pleomorphic salivary adenoma; qMSP=quantitative methylation-specific real-time polymerase chain reaction; WT1=Wilms’ tumour 1.

qMSP results for each sample by tumour suppressor gene. Each result is referenced to the artificially 5% methylation control sample (RQ=2^−ΔΔCT^).

**Table 4:** Performance of single promoter methylation as biomarkers of malignant transformation of PSA to Ca ex PSA

	**hTERT**	**WT1**	**RASSF1**	**p16**	**RAR*β***	**CYGB**	**TMEFF2**
Sensitivity (%)	12.9	9.7	51.6	12.9	0	0	0
Specificity (%)	100	100	92.9	100	100	100	100
Difference between benign and malignant (Fisher's exact)	*P*=0.11	*P*=0.24	*P*<0.001	*P*=0.11	—	—	—

Abbreviations: Ca ex PSA=carcinoma ex pleomorphic adenoma; hTERT=human telomerase reverse transcriptase; PSA=pleomorphic salivary adenoma; WT1=Wilms’ tumour 1.

Sensitivity and specificity for each tumour suppressor gene as a test for malignancy in a sample.
